# Supplementary material for: Pushed to extremes: distinct effects of high temperature versus pressure on the structure of STEP
Source: Commun Biol. 2024 Jan 12;7:59. doi: 10.1038/s42003-023-05609-0 (PMC10786866; doi:10.1038/s42003-023-05609-0)
Supplement: Supplementary file 1 — Supplementary Table and Figures [file 42003_2023_5609_MOESM1_ESM.pdf]

Supplementary Information

Guerrero*, Ebrahim*, et al. (2023) "Pushed to extremes: distinct effects of high temperature versus pressure on the structure of STEP"

	LoTP	HiT	HiP
# waters, deposited	149	69	98
# waters, automated with truncated data	141	69	93

Supplementary Table 1: Waters are sensitive to perturbations but robust to modeling method. See Methods for details of structure factor data truncation and automated water placement.

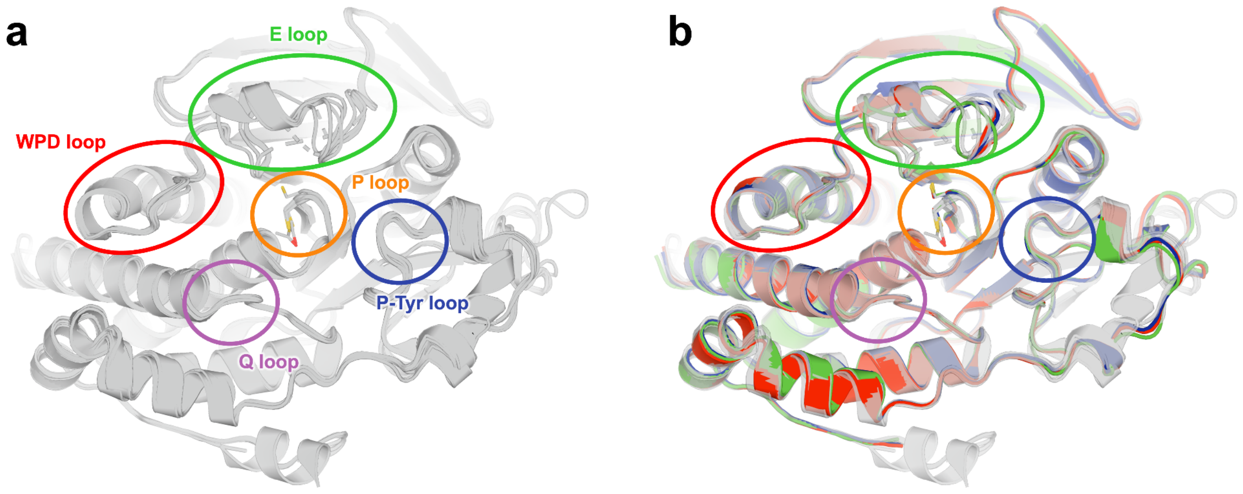

Supplementary Figure 1: Superposition of our new STEP structures and all previous STEP structures.

a) All previous human and mouse structures overlaid: PDB IDs 2bv5, 2bij, 2cjz, 5ovr, 5ovx, 5ow1, 6h8r, and 6h8s (gray).
b) Our LoTP (blue), HiT (red), and HiP (green) structures are shown overlaid with all previous human and mouse STEP structures.

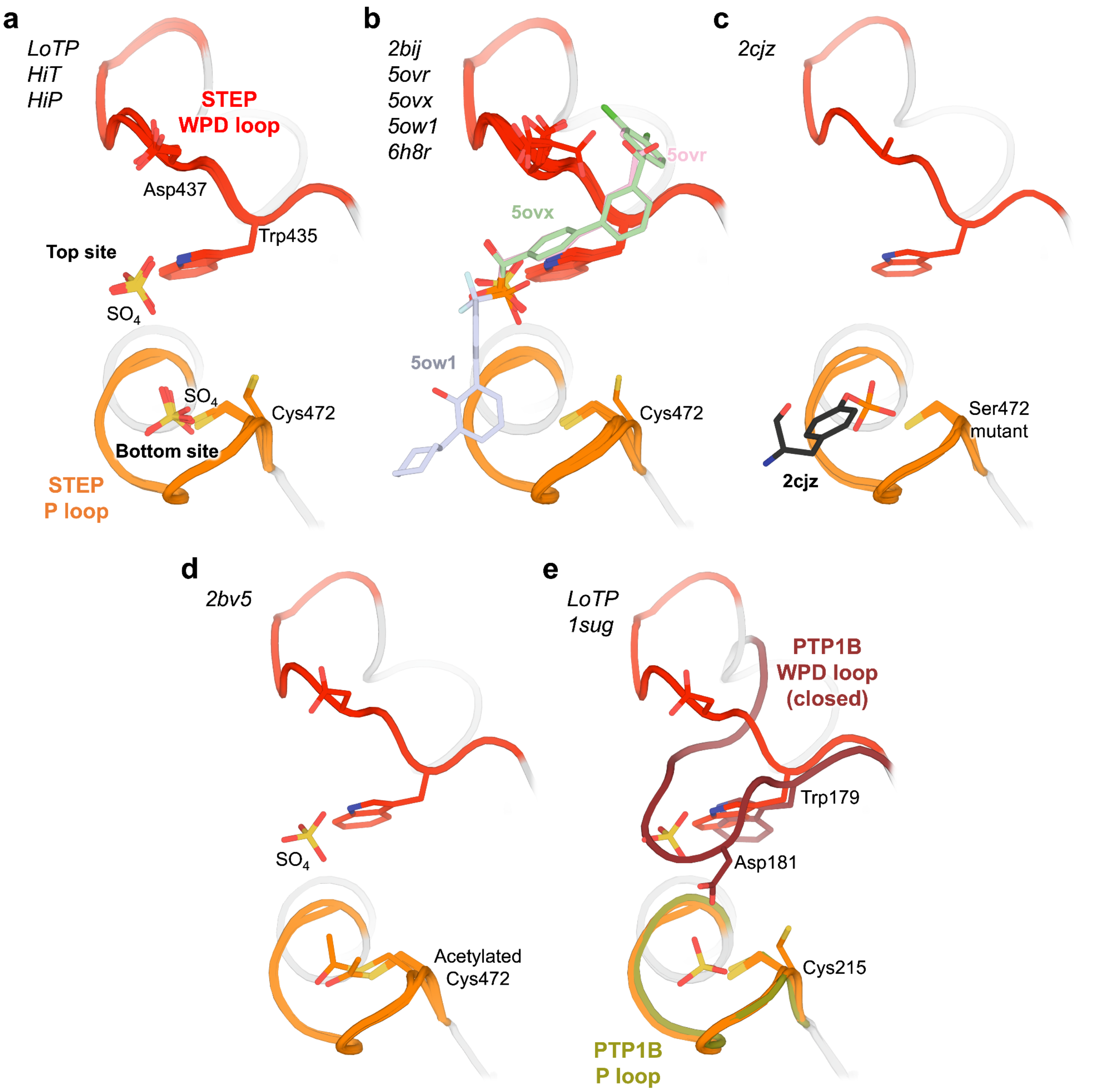

Supplementary Figure 2: Previous STEP structures did not have two sulfates in the active site.

a) Sulfates in our LoTP, HiT, and HiP structures in both the “top” and “bottom” sites. Key residues in the WPD loop and the P loop are labeled.
b) A sulfate or analogous group from a competitive inhibitor only in the top site, in PDB ID 2bij, 5ovr, 5ovx, 5ow1, and 6h8r.
c) A phosphate group in a pTyr substrate in PDB ID only in the bottom site, in PDB ID 2cjz.
d) A sulfate in the top site and an acetylated catalytic Cys472 in the bottom site, in PDB ID 2bv5.
e) Superposition of the PTP1B closed state from PDB ID 1sug [73] with our STEP LoTP structure, illustrating that the closed WPD loop of PTP1B aligns well with the top sulfate in STEP. Key residues from PTP1B in the WPD loop and P loop are labeled.

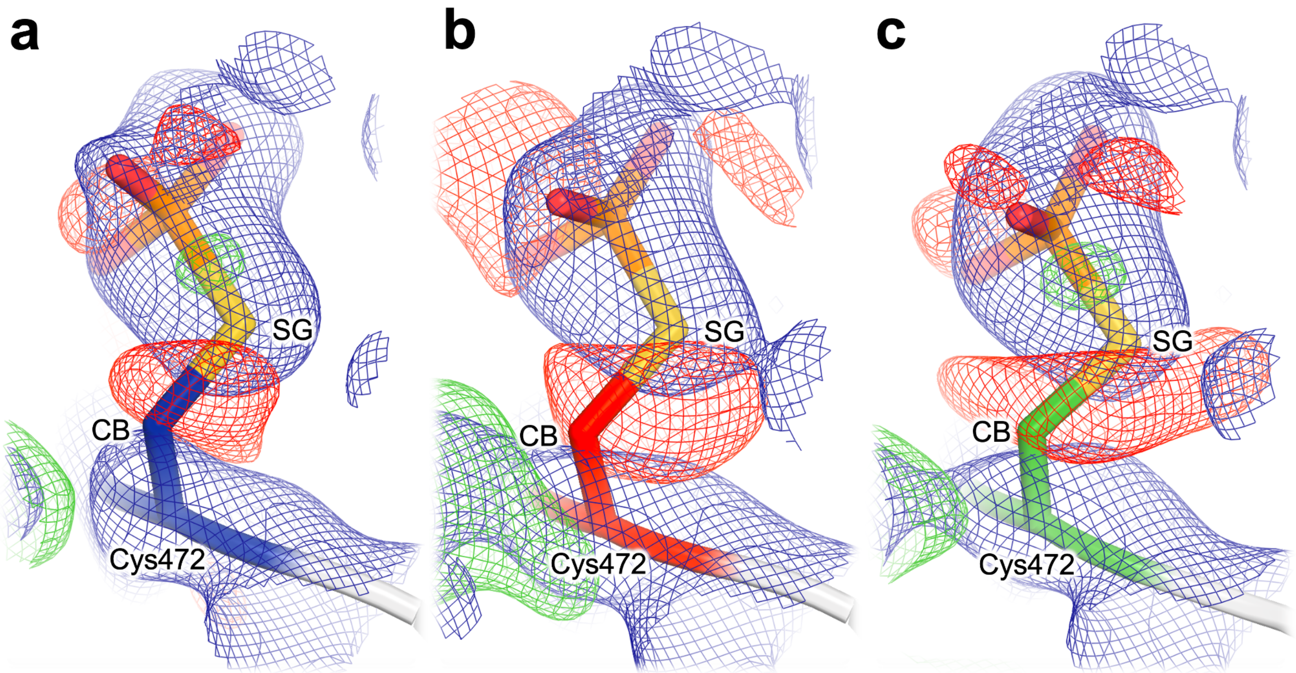

Supplementary Figure 3: Phosphocysteine is a poor fit for the catalytic Cys472.
Refinement of models with a putative phosphocysteine intermediate covalently bound to the catalytic Cys472 result in reasonable fit to parts of the 2Fo-Fc (blue mesh, 1.0 σ) electron density, but unacceptable fits to the Fo-Fc difference electron density (green mesh, +3.0 σ; red mesh, -3.0 σ).

a) LoTP.
b) HiP.
c) HiT.

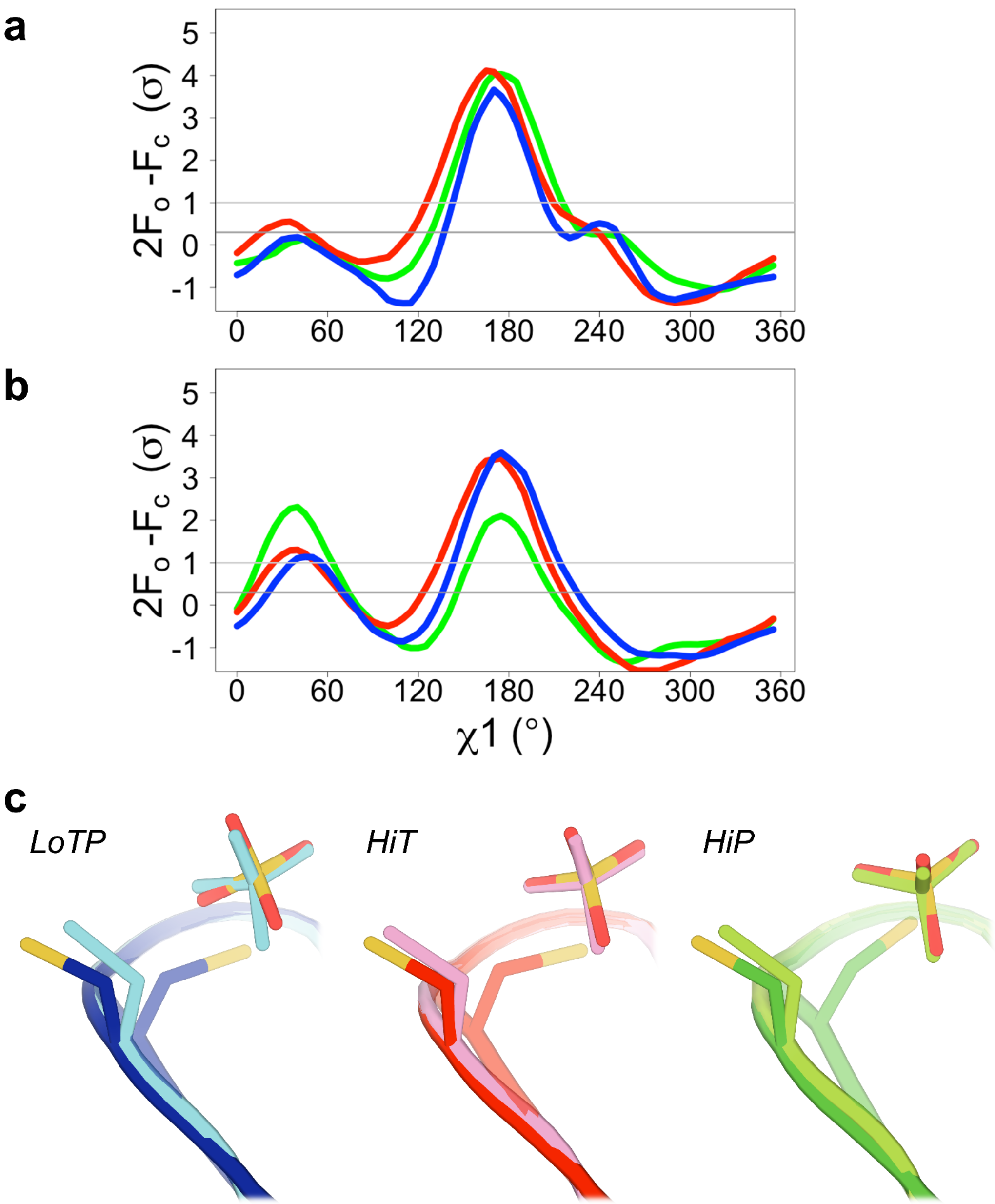

Supplementary Figure 4: Ringer analysis of the catalytic cysteine.

a) Ringer curve for Cys472 using input model prepared by extracting alternate conformation A (primary rotamer, pointed away from sulfate) from the dual-conformation model and performing reciprocal-space refinement in PHENIX.

b) Ringer curve for Cys472 using input model prepared by extracting alternate conformation A from the dual-conformation model, truncating to Cβ, performing real-space refinement in Coot, and restoring the rest of the side chain with the standard library rotamer closest to the original alternate conformation A in Coot, with no further refinement. This strategy aims to bypass the constraints on backbone positioning imposed by particular side-chain rotamers during refinement. The data for generating a) and b) curves is available in **Supp. Data 3**.

c) Models from a–b), demonstrating differences in Cα-Cβ vectors, which can influence Ringer results. *Left:* LoTP (blue), *middle:* HiT (red), *right:* HiP (green). Shown in each panel are alternate conformation A from a) (darkest color), adjusted input conformation from b) (intermediate color), and alternate conformation B for visual reference (lightest color).

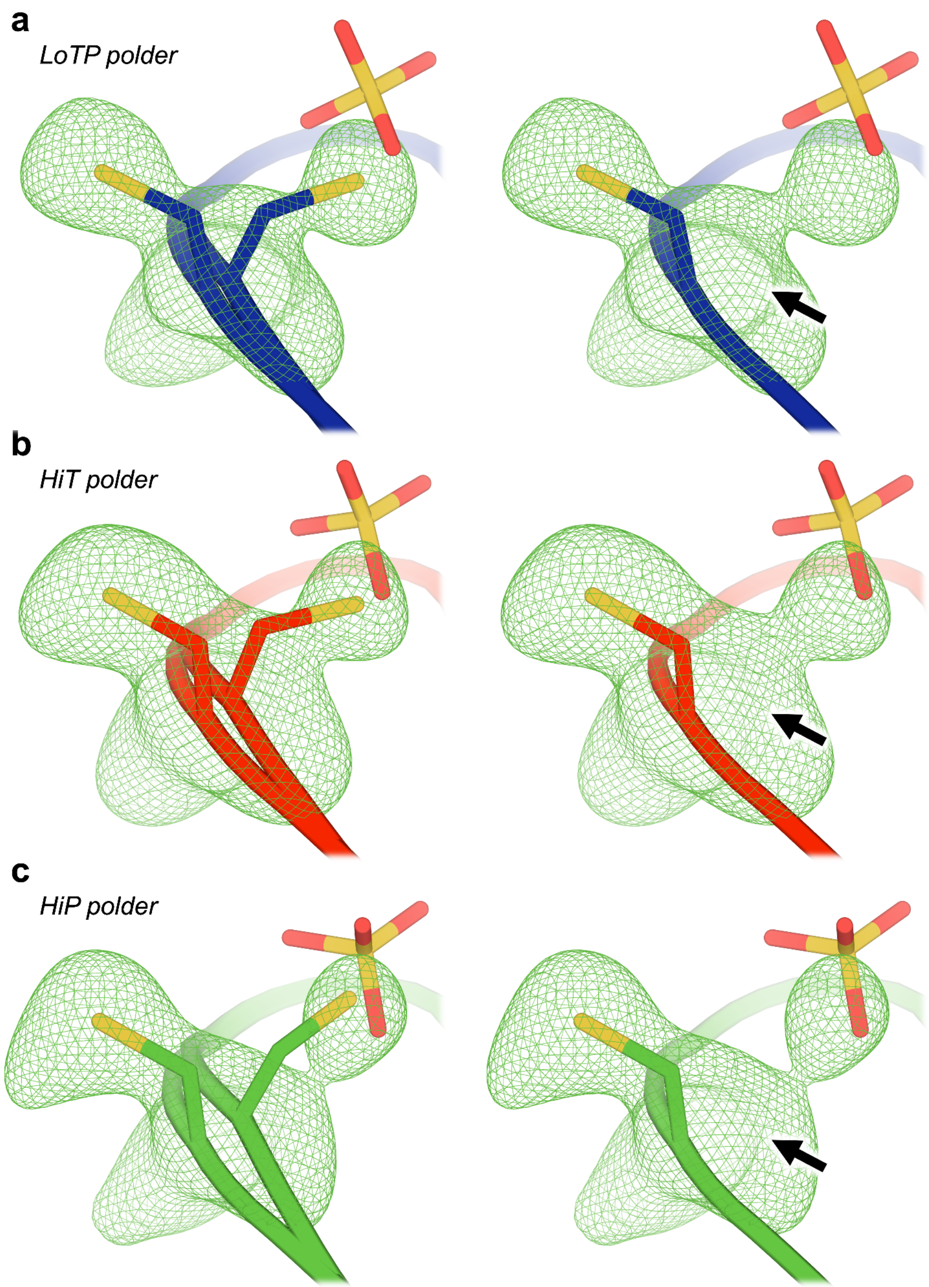

Supplementary Figure 5: Polder maps for the catalytic cysteine.
Left: Our dual-conformation model of Cys472 is supported by a Polder map (5 σ), including the backbone Cα atom (arrow).
Right: A putative single-conformation model for Cys472, obtained by extracting only the primary conformation from our dual-conformation model, highlights Polder density consistent with a missing secondary conformation, particularly noticeable for the backbone including the Cα atom (arrow).

a) LoTP.
b) HiT.
c) HiP.

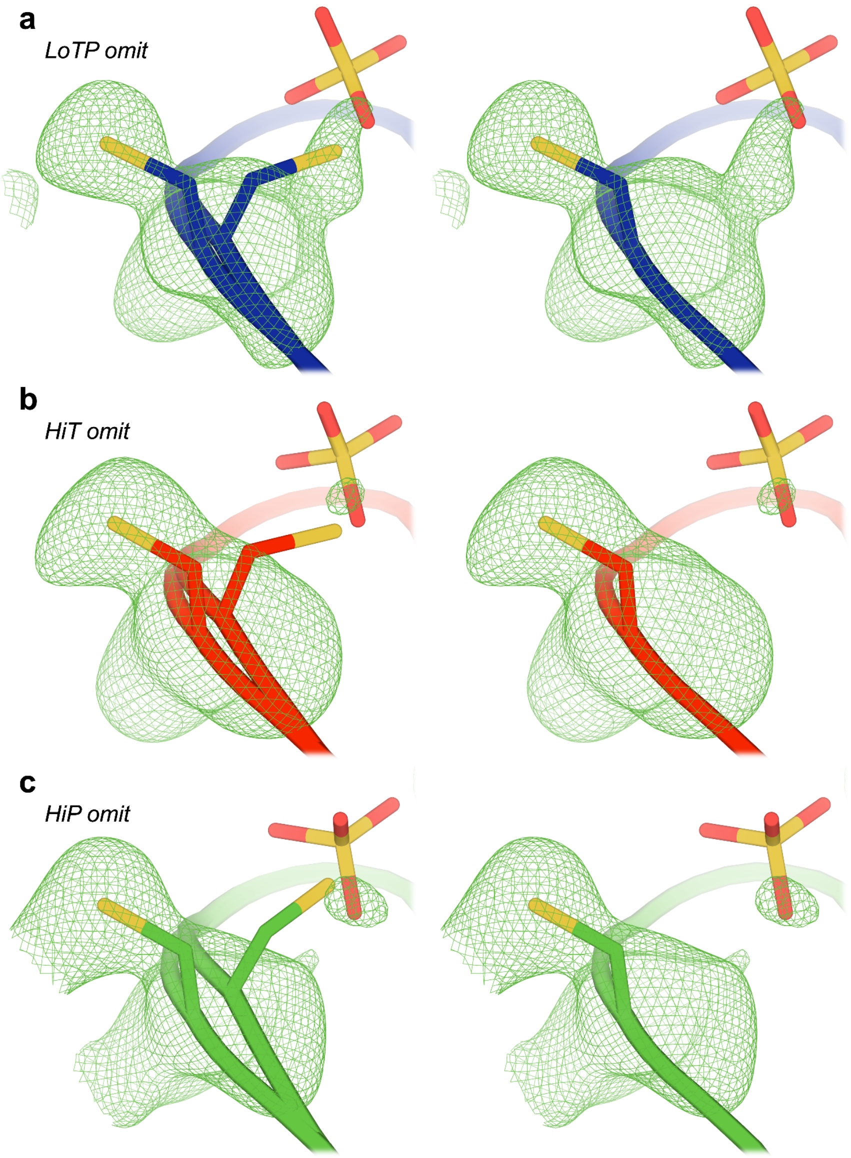

Supplementary Figure 6: Omit maps for the catalytic cysteine.
Same as **Supp. Fig. 5**, but using omit maps (3 σ) instead of Polder maps.

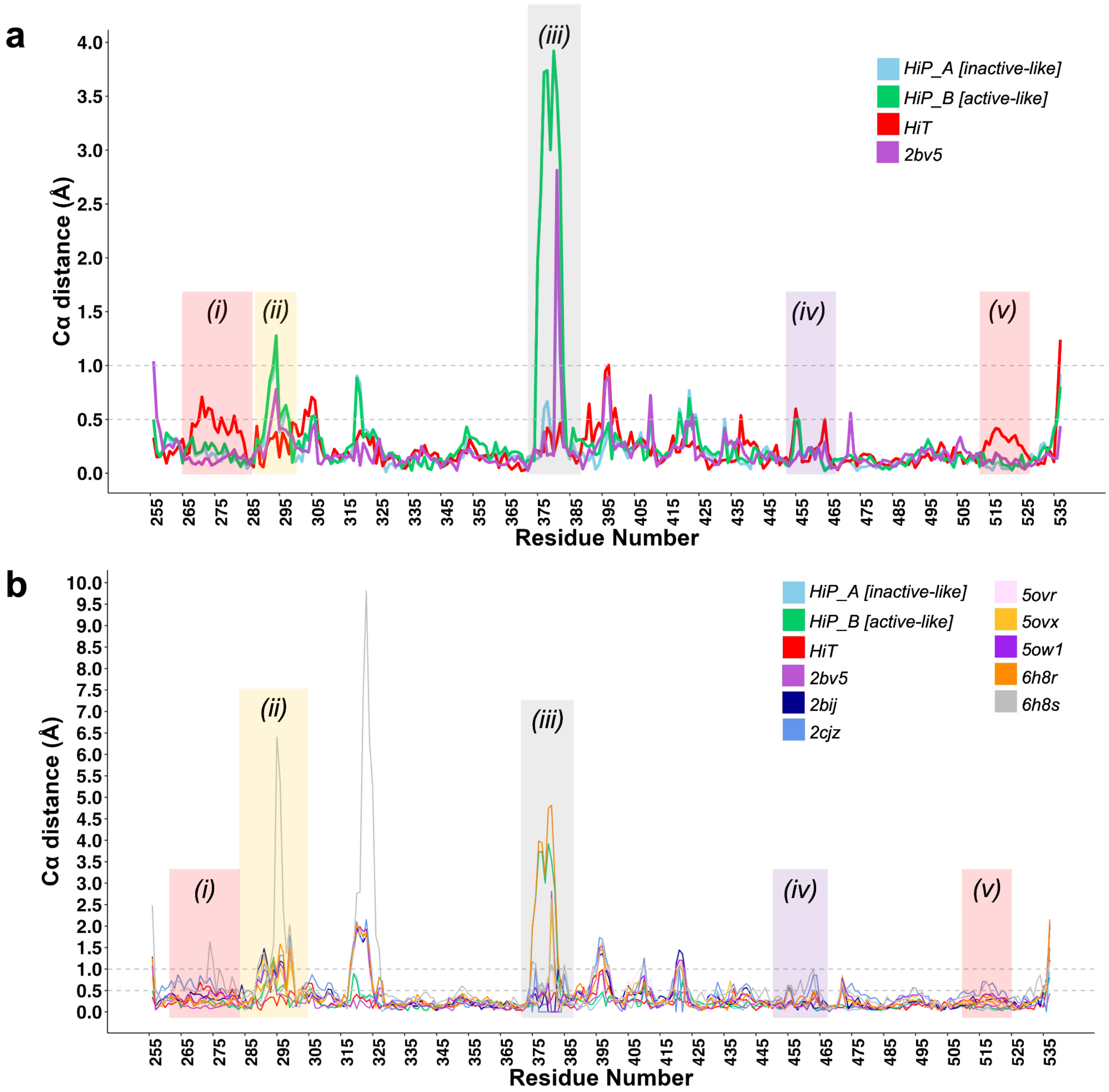

Supplementary Figure 7: Global backbone displacements due to high temperature vs. pressure, relative to previous STEP structures.

a) Cα distances for the HiT and HiP structures relative to the reference LoTP structure vs. amino acid sequence, including the only previous STEP structure in the same crystal form as our structures (PDB ID 2bv5).

b) Same as (a), but also including all previous human and mouse STEP structures that have a different crystal form (PDB ID 2bij, 2cjz, 5ovr, 5ovx, 5ow1, 6h8r, 6h8s). The data for generating these graphs is available in **Supp. Data 1**.

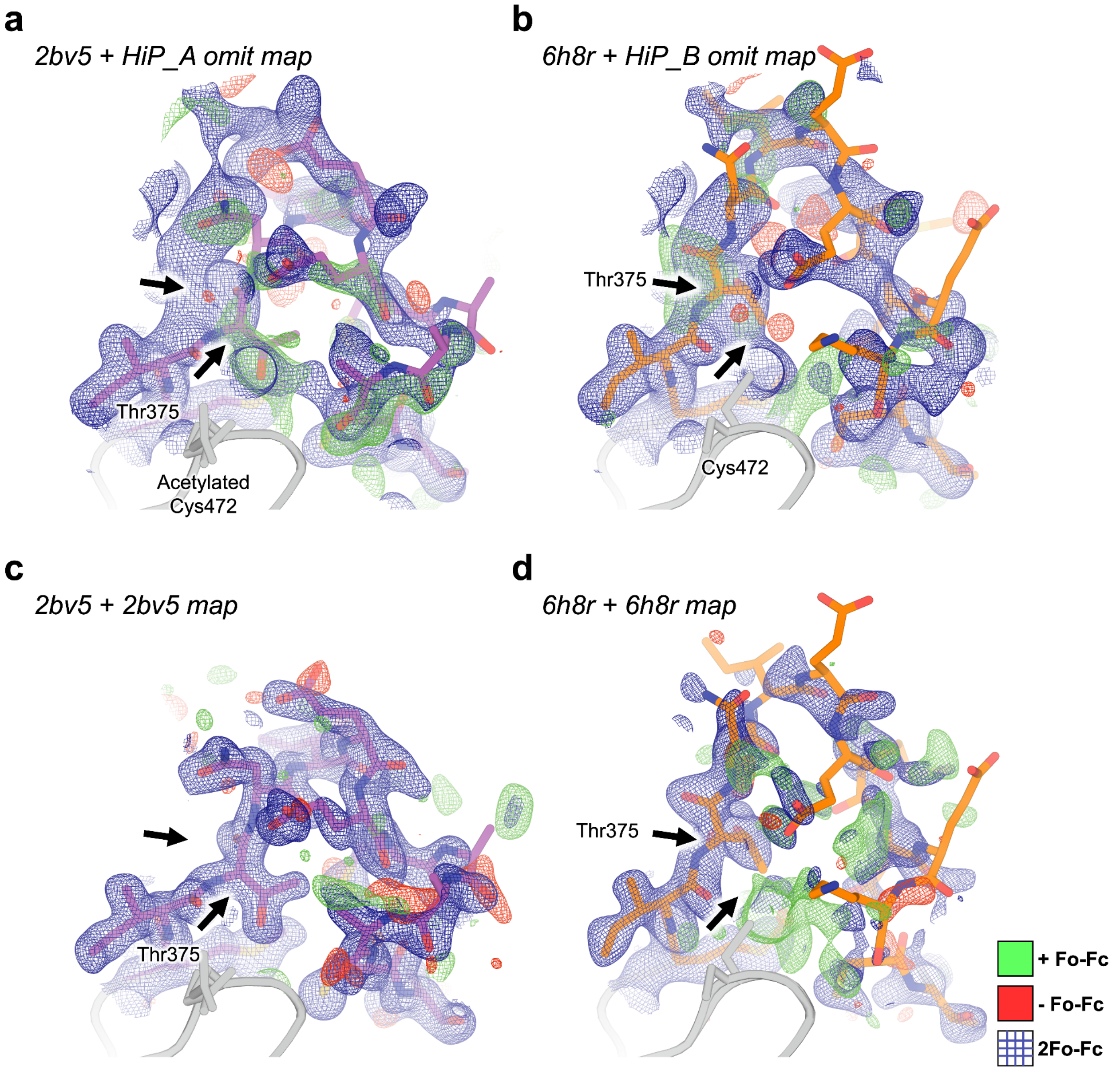

Supplementary Figure 8: The E loop at high pressure samples two distinct previously observed states.

a) PDB 2bv5 model with our HiP 2Fo-Fc (contoured at 1 σ) and Fo-Fc (contoured at ±3 σ) maps omitting alternate conformation A.

b) PDB 6h8r model with our HiP 2Fo-Fc and Fo-Fc maps omitting alternate conformation B.

c) 2bv5 model with 2bv5 2Fo-Fc and Fo-Fc maps.

d) 6h8r model with 6h8r 2Fo-Fc and Fo-Fc maps.

Arrows highlight the locations of the dual-conformation Thr375 in our HiP model, for reference.

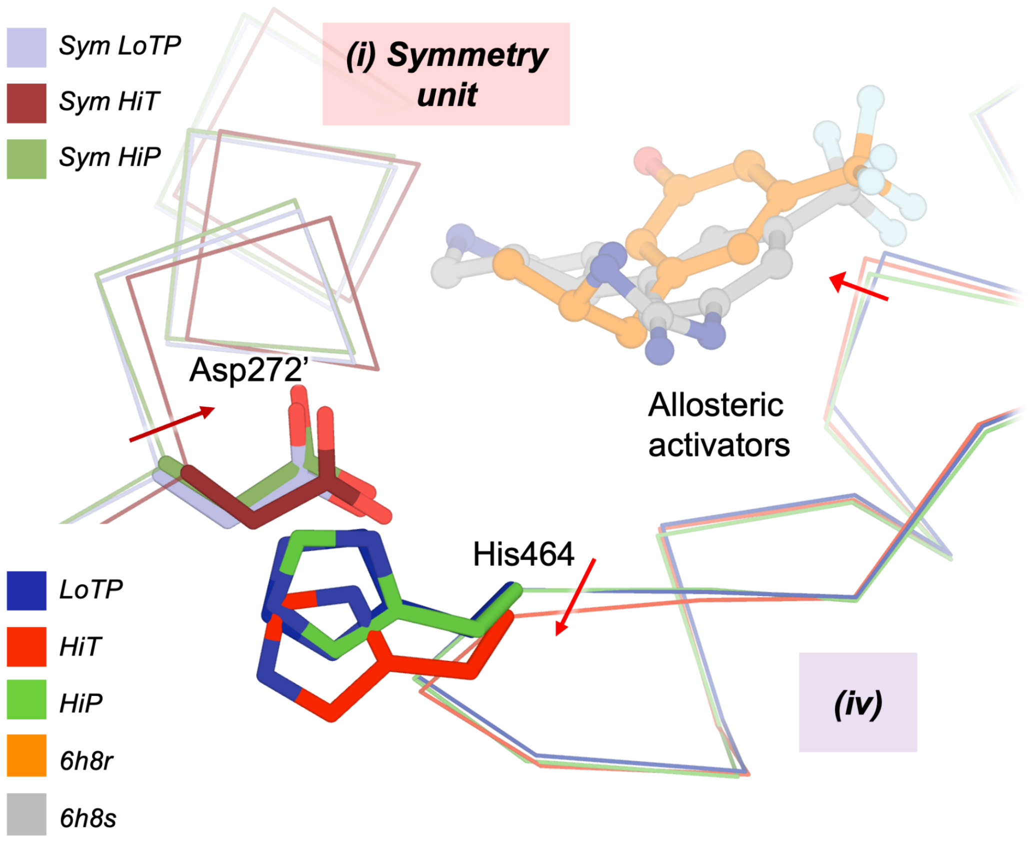

Supplementary Figure 9: Backbone displacements at the allosteric activator site due to high temperature.
Zoom-in of the area including region (iv) (residues 454-467, S loop) from **Fig. 3a**. The small-molecule allosteric activators from PDB ID 6h8r (orange) and 6h8s (gray) are shown for context, as well as the symmetry-related Asp272' from region (i) from **Fig. 3a**.

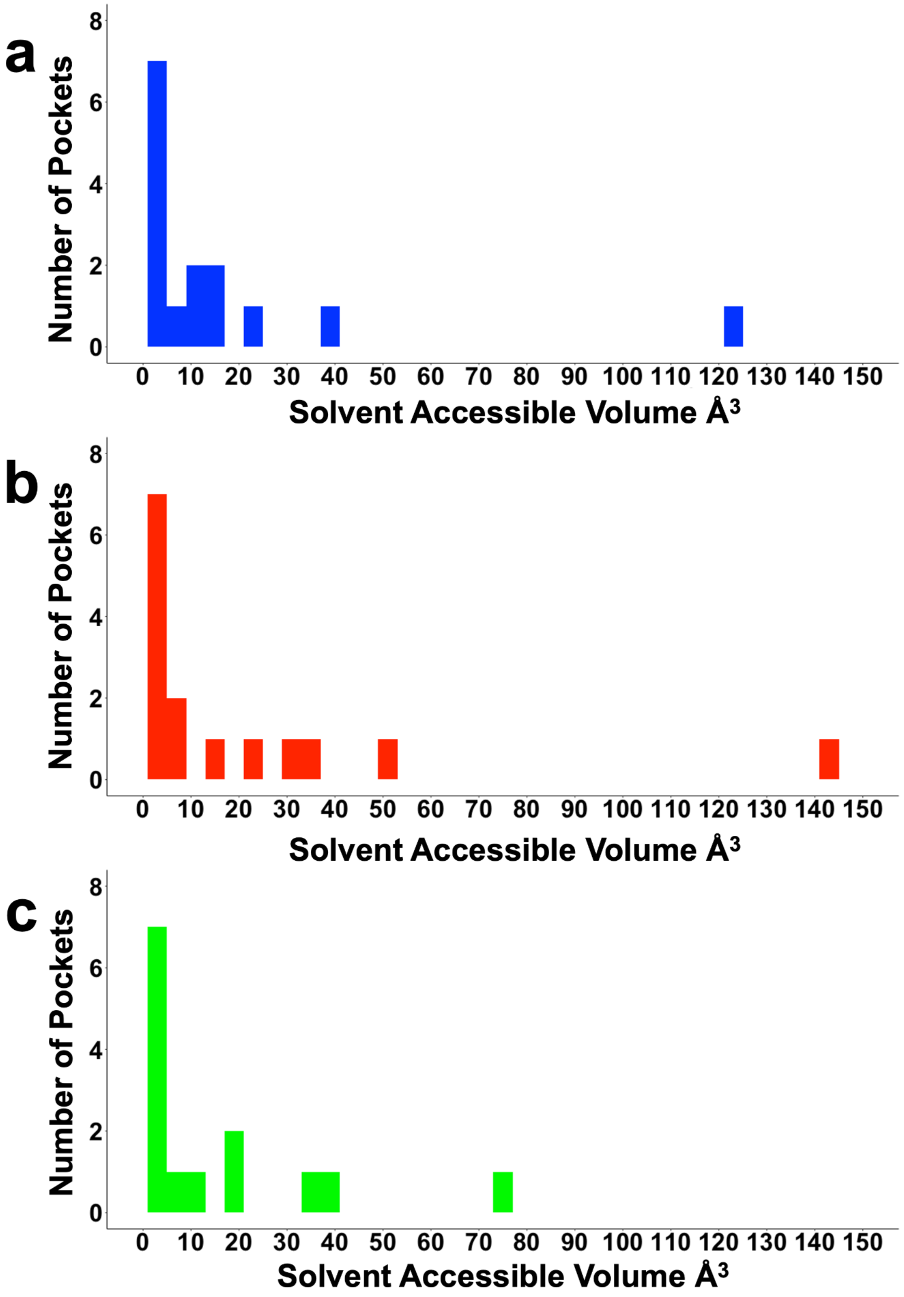

Supplementary Figure 10: Distribution of pocket/cavity volumes for each structure.
Histograms of solvent-accessible pocket volume from CASTp [50].

a) LoTP (33 pockets).
b) HiT (28 pockets).
c) HiP (36 pockets). The data for generating these graphs is available in **Supp. Data 4**.

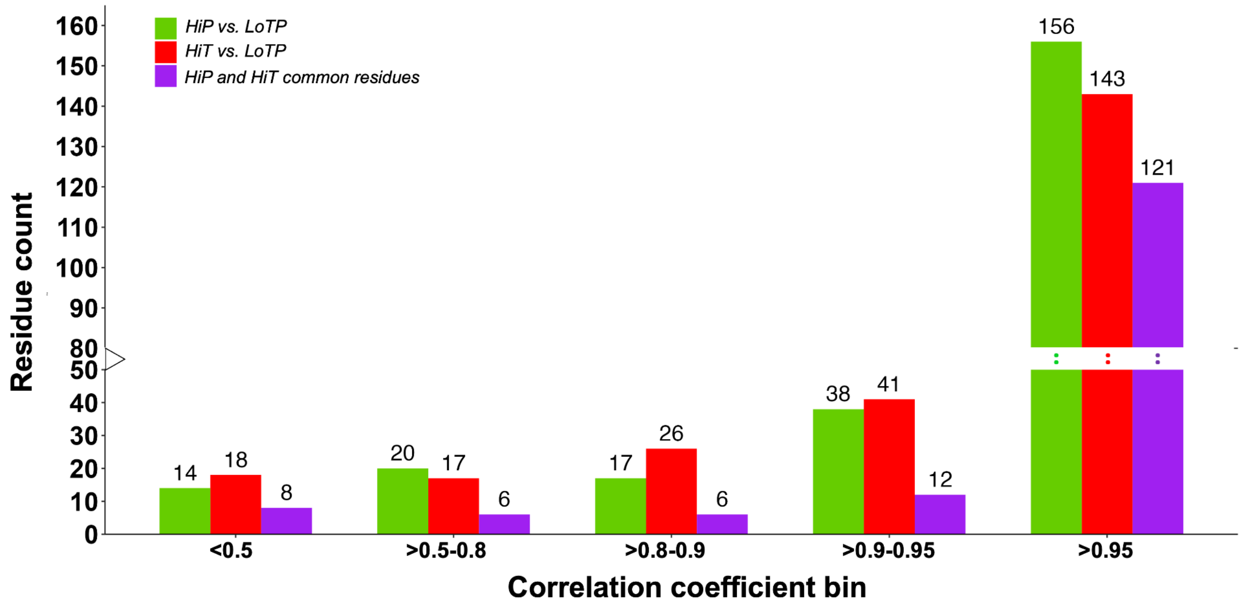

Supplementary Figure 11: Ringer analysis points to widespread but distinct effects of high temperature vs. pressure on side-chain conformations.

For each residue in STEP (excluding the flexible E loop), Ringer curves were calculated for HiT, HiP, and LoTP, and Pearson correlation coefficient (CC) was calculated relative to LoTP. Shown are the number of residues with given Ringer CC values for HiT, for HiP, and for both HiT + HiP. The data for generating this graph is available in **Supp. Data 2**.

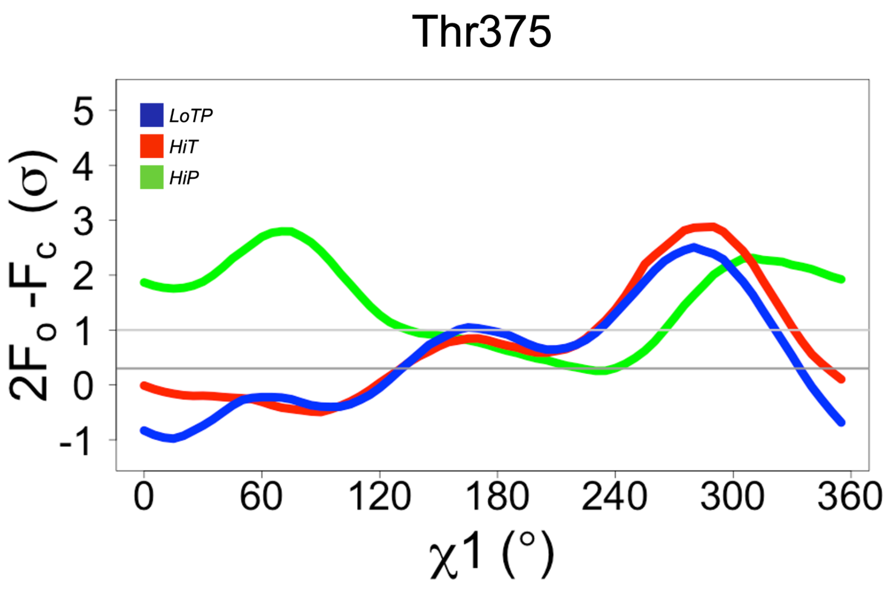

Supplementary Figure 12: Differences in Ringer curves at high pressure due to distinct backbone positioning.

Thr375 has similar χ1 peaks for LoTP and HiT, but different χ1 peaks for HiP when starting from the alternate E-loop backbone conformation. (Note that Thr generally has two χ1 peaks even with a single conformation because it is a β-branched side chain with two different γ atoms.) This is associated with backbone shifts starting at Thr375 at the beginning of the E loop that occur only at HiP. LoTP in blue, HiT in red, HiP in green, as in **Fig. 5**. The data for generating this graph is available in **Supp. Data 2**.
