# Supplementary material for: Pushed to extremes: distinct effects of high temperature versus pressure on the structure of STEP
Source: Commun Biol. 2024 Jan 12;7:59. doi: 10.1038/s42003-023-05609-0 (PMC10786866; doi:10.1038/s42003-023-05609-0)
Supplement: Supplementary file 2 — Description of Additional Supplementary Files [file 42003_2023_5609_MOESM2_ESM.pdf]

Description of Additional Supplementary Files

File name: Supplementary Data 1
Description: The source data behind the Fig. 3a and Supp. Fig.7.

File name: Supplementary Data 2
Description: The source data behind Fig. 5, Supp. Fig. 11 and Supp. Fig. 12

File name: Supplementary Data 3
Description: The source data behind the Supp. Fig. 4

File name: Supplementary Data 4
Description: The source data behind the Supp. Fig. 10
