# Supplementary material for: Pushed to extremes: distinct effects of high temperature versus pressure on the structure of STEP
Source: Commun Biol. 2024 Jan 12;7:59. doi: 10.1038/s42003-023-05609-0 (PMC10786866; doi:10.1038/s42003-023-05609-0)
Supplement: Supplementary file 7 — Reporting Summary [file 42003_2023_5609_MOESM7_ESM.pdf]

nature portfolio

Corresponding author(s): Daniel A. Keedy

Last updated by author(s): Nov 8, 2023



# Reporting Summary

Nature Portfolio wishes to improve the reproducibility of the work that we publish. This form provides structure for consistency and transparency in reporting. For further information on Nature Portfolio policies, see our Editorial Policies and the Editorial Policy Checklist.

## Statistics

For all statistical analyses, confirm that the following items are present in the figure legend, table legend, main text, or Methods section.

| n/a | Confirmed | |
|---|---|---|
| ☒ | ☐ | The exact sample size (*n*) for each experimental group/condition, given as a discrete number and unit of measurement |
| ☒ | ☐ | A statement on whether measurements were taken from distinct samples or whether the same sample was measured repeatedly |
| ☒ | ☐ | The statistical test(s) used AND whether they are one- or two-sided<br>*Only common tests should be described solely by name; describe more complex techniques in the Methods section.* |
| ☒ | ☐ | A description of all covariates tested |
| ☒ | ☐ | A description of any assumptions or corrections, such as tests of normality and adjustment for multiple comparisons |
| ☒ | ☐ | A full description of the statistical parameters including central tendency (e.g. means) or other basic estimates (e.g. regression coefficient) AND variation (e.g. standard deviation) or associated estimates of uncertainty (e.g. confidence intervals) |
| ☒ | ☐ | For null hypothesis testing, the test statistic (e.g. *F*, *t*, *r*) with confidence intervals, effect sizes, degrees of freedom and *P* value noted<br>*Give P values as exact values whenever suitable.* |
| ☒ | ☐ | For Bayesian analysis, information on the choice of priors and Markov chain Monte Carlo settings |
| ☒ | ☐ | For hierarchical and complex designs, identification of the appropriate level for tests and full reporting of outcomes |
| ☒ | ☐ | Estimates of effect sizes (e.g. Cohen's *d*, Pearson's *r*), indicating how they were calculated |

*Our web collection on statistics for biologists contains articles on many of the points above.*

## Software and code

Policy information about availability of computer code

| | |
|---|---|
| Data collection | Data were collected using the ADX software suite on beamline ID7B2 at Cornell High Energy Synchrotron Source |
| Data analysis | DIALS v3.5.0 and DIMPLE v2.6.1 were used for data reduction. PHENIX v1.19.2 and CCP4 v8.0 were used for data refinement. Coot v0.9.8.7 was used for iterative model building. PHENIX v1.19.2 was used for model and rotamer analysis. Ringer 2.0 was used for rotamer analysis. Plots were generated using ggplot v3.4.2 and ggbreak v0.1.1. Calpha distances were calculated using VMD v1.9.4. RoPE and ProteinVolume v1.3 were used to assess protein models. |

For manuscripts utilizing custom algorithms or software that are central to the research but not yet described in published literature, software must be made available to editors and reviewers. We strongly encourage code deposition in a community repository (e.g. GitHub). See the Nature Portfolio guidelines for submitting code & software for further information.

## Data

Policy information about availability of data

All manuscripts must include a data availability statement. This statement should provide the following information, where applicable:

- Accession codes, unique identifiers, or web links for publicly available datasets
- A description of any restrictions on data availability
- For clinical datasets or third party data, please ensure that the statement adheres to our policy

Coordinates and structure factors that were generated during the course of this study have been deposited in the Protein Data Bank with the accession codes 8SLS

(STEP at cryogenic temperature and ambient pressure), 8SLT (STEP at physiological temperature and ambient pressure), and 8SLT (STEP at cryogenic temperature and high pressure). The protein structure used as a search model for molecular replacement is accessible in the Protein Data Bank under accession codes 2BV5.

## Research involving human participants, their data, or biological material

Policy information about studies with human participants or human data. See also policy information about sex, gender (identity/presentation), and sexual orientation and race, ethnicity and racism.

| | |
|---|---|
| Reporting on sex and gender | Information not collected. Our study doesn't pertain to research involving human participants, their data, or biological material. |
| Reporting on race, ethnicity, or other socially relevant groupings | Information not collected. Our study doesn't pertain to research involving human participants, their data, or biological material. |
| Population characteristics | Information not collected. Our study doesn't pertain to research involving human participants, their data, or biological material. |
| Recruitment | Information not collected. Our study doesn't pertain to research involving human participants, their data, or biological material. |
| Ethics oversight | Information not collected. Our study doesn't pertain to research involving human participants, their data, or biological material. |

Note that full information on the approval of the study protocol must also be provided in the manuscript.

# Field-specific reporting

Please select the one below that is the best fit for your research. If you are not sure, read the appropriate sections before making your selection.

☒ Life sciences ☐ Behavioural & social sciences ☐ Ecological, evolutionary & environmental sciences

For a reference copy of the document with all sections, see nature.com/documents/nr-reporting-summary-flat.pdf

# Life sciences study design

All studies must disclose on these points even when the disclosure is negative.

| | |
|---|---|
| Sample size | No sample size calculation was performed. A sample size of 3 data collection conditions (cryogenic temperature, ambient pressure; physiological temperature, ambient pressure; cryogenic temperature, high pressure) was chosen. We deem two avant-garde crystallographic data collection methods sufficient to assess heterogeneity in protein structures compared to the typical crystallographic experiment. |
| Data exclusions | No data were excluded. |
| Replication | To verify the reproducibility of the experimental findings, our data were reviewed and assessed in meetings between the research team involved in producing the manuscript. Our data and findings have also been presented at a number of seminars and international conferences of our peers. Findings were not replicated. We do have a number of replicate crystallographic datasets, however those presented in our paper were selected for analysis due to their superior data quality statistics. |
| Randomization | Randomization is not relevant to our study. We are comparing the effects of different data collection conditions on protein crystal structures, which does not involve randomization of samples, organisms, or participants. |
| Blinding | Blinding is not relevant to our study. We are comparing the effects of different data collection conditions on protein crystal structures, which does not involve blinding of investigators to group allocation during date collection and/or analysis. |

# Reporting for specific materials, systems and methods

We require information from authors about some types of materials, experimental systems and methods used in many studies. Here, indicate whether each material, system or method listed is relevant to your study. If you are not sure if a list item applies to your research, read the appropriate section before selecting a response.

## Materials & experimental systems

| n/a | Involved in the study |
| --- | --- |
| ☒ | ☐ Antibodies |
| ☒ | ☐ Eukaryotic cell lines |
| ☒ | ☐ Palaeontology and archaeology |
| ☒ | ☐ Animals and other organisms |
| ☒ | ☐ Clinical data |
| ☒ | ☐ Dual use research of concern |
| ☒ | ☐ Plants |

## Methods

| n/a | Involved in the study |
| --- | --- |
| ☒ | ☐ ChIP-seq |
| ☒ | ☐ Flow cytometry |
| ☒ | ☐ MRI-based neuroimaging |
